# Improving Team-Based Decision Making Using Data Analytics and Informatics: Protocol for a Collaborative Decision Support Design

**DOI:** 10.2196/16047

**Published:** 2019-11-27

**Authors:** Don Roosan, Anandi V Law, Mazharul Karim, Moom Roosan

**Affiliations:** 1 Western University of Health Sciences College of Pharmacy Pomona, CA United States; 2 Chapman University School of Pharmacy Irvine, CA United States

**Keywords:** informatics, health care team, data science, decision support techniques, decision-making, computer-assisted, data display, diagnosis, computer-assisted

## Abstract

**Background:**

According to the September 2015 Institute of Medicine report, Improving Diagnosis in Health Care, each of us is likely to experience one diagnostic error in our lifetime, often with devastating consequences. Traditionally, diagnostic decision making has been the sole responsibility of an individual clinician. However, diagnosis involves an interaction among interprofessional team members with different training, skills, cultures, knowledge, and backgrounds. Moreover, diagnostic error is prevalent in the interruption-prone environment, such as the emergency department, where the loss of information may hinder a correct diagnosis.

**Objective:**

The overall purpose of this protocol is to improve team-based diagnostic decision making by focusing on data analytics and informatics tools that improve collective information management.

**Methods:**

To achieve this goal, we will identify the factors contributing to failures in team-based diagnostic decision making (aim 1), understand the barriers of using current health information technology tools for team collaboration (aim 2), and develop and evaluate a collaborative decision-making prototype that can improve team-based diagnostic decision making (aim 3).

**Results:**

Between 2019 to 2020, we are collecting data for this study. The results are anticipated to be published between 2020 and 2021.

**Conclusions:**

The results from this study can shed light on improving diagnostic decision making by incorporating diagnostics rationale from team members. We believe a positive direction to move forward in solving diagnostic errors is by incorporating all team members, and using informatics.

**International Registered Report Identifier (IRRID):**

DERR1-10.2196/16047

## Introduction

### Background

Americans experience at least one diagnostic error in their lifetime, sometimes with devastating consequences (Institute of Medicine [IOM] report 2015). Lack of timely attention to diagnostic error can have dire implications for public health, as exemplified by the widely reported diagnostic error regarding Ebola virus infection in a Dallas hospital emergency department (ED) [1]. Diagnostic error is likely to be one of the most common types of errors in ED settings [2]. The high-paced, high-volume, low-certainty, multiagent, dynamic, and complex environment may lead to diagnostic errors and adverse events [3-6]. Thus, in an environment prone to interruptions, vital patient information and cues to make a diagnosis are often lost during information collection and integration among physicians, residents, nurses, and other health care providers.

The team-based diagnostic approach has the potential to reduce errors. Although the current diagnostic process is often the responsibility of an individual clinician, ideally the diagnostic process involves collaboration among multiple health care professionals [7]. To manage the increasing complexity, clinicians will need to collaborate effectively and draw upon the knowledge and expertise of other health care professionals. Collaborative problem solving has been found to have a positive impact on diagnostic performance for team members to combine, sort, and filter new information [8-11]. Current diagnostic decision support tools do not support team-based decision making. These tools can generate diagnostic hypotheses based on the information already entered into the electronic health record (EHR) [12-15]. However, information loss in ED is more common during team communication among health care professionals [16,17]. Therefore, the recent IOM report calls for research into the process of how, where, when, and who is responsible for the entry of the vital information into the system to understand the etiology of failures in the team-based diagnostic decision-making process [18].

Research has shown that technology can positively impact provider interactions and coordination, helping group dynamics and efficiency [19]. Various computer supported cooperative work studies in health care have shown that clinicians deploy working records or provisional information to facilitate team collaboration, mostly in paper environments during case discussions to exchange key information [20-22]. These working records are essentially summaries of patients’ situations or important information cues that providers write down during patient interviews or during the information-gathering stage. Currently, the information documented on these working records is not transferred to the EHR and often is discarded after knowledge sharing sessions. Moreover, the decision support tools in the EHR do not support such computerized transitional documentation [23]. For example, nurses in ED collect patient medical history into transitional documents. Clinicians enter patient interview information related to diagnosis on paper or sticky notes [23]. However, the informal information, if shared with the team, can help to achieve shared team situation awareness to reach the correct diagnosis [24]. Research on collaborative environments has shown that sharing a physical workspace to communicate information can provide benefits such as improved activity awareness and coordination [25-28]. For example, Defense Collaboration Services (developed by the US Department of Defense) have shared Web-based platforms that can be accessed by different team members, and they can raise information need as well as input vital information cues related to mission planning [29,30]. Such real-time platforms in health care can provide an overview of the patient’s situation from different information-gathering agents (eg, nurses, residents, students, and physicians) to reach the correct diagnosis.

### Objective

The informal information in a real-time workspace can help the team to communicate and interpret vital information with each other, which can improve team-based diagnostic decision making in the ED by reducing the loss of information. The objective of our study is to develop a collaborative prototype for improving team diagnostic decision making using an informatics approach.

## Methods

### Overview

We want to focus on all types of diagnosis for adult patients who come to the ED in both the trauma and medicine units. This will ensure that we can generalize the future prototype for all ED patients. The overall methodology is described in the following 3 aims.

- Aim 1: identify factors contributing to failures in team-based diagnostic decision making
- Aim 2: understand the barriers in using health information technology (IT) tools for team collaboration
- Aim 3: design and evaluate a collaborative decision-making prototype

### Aim 1: Identify Factors Contributing to Failures in Team-Based Diagnostic Decision Making

The research questions are as follows: (1) What are the specific diagnostic workflow processes that are vulnerable to failures in information gathering, integrating, interpreting, and establishing an explanation of the correct diagnosis? and (2) What specific information cues do teams share with each other to reach a diagnosis collaboratively?

#### Aim 1 Methods Overview

We will use the combination of direct observation, hierarchical task analysis (HTA), and health care failure mode and effect analysis (HFMEA) to analyze team tasks in the diagnosis process [31-33]. HTA involves describing the task being analyzed through the breakdown of the task into a hierarchy of goals, subgoals, operations, and plans [31]. The HFMEA technique will help us detect possible failure modes of each of the subprocesses and identify potential causes, effects, and solutions for the failure in the team diagnostic process [32]. A research assistant with qualitative coding background will analyze the data. The steps involved in this method are as follows:

- Step 1: observe scenarios in ED settings and transcribe the scenarios from audio recordings
- Step 2: use data from the transcription to create HTA process maps
- Step 3: conduct HFMEA to identify failures and improvement strategies

#### Step 1: Observe Scenarios

The observation will start once the patient is admitted in the ED. A total of 2 research assistants will simultaneously observe the ED nurse and the attending physician. The observations will be nonintrusive, and researchers will turn on audio recorders only when the team is discussing or communicating with each other regarding the patient case [34-40]. We will take notes and audio record the conversation, interactions, and case discussion among the ED team members. We will transcribe the audio recordings and collect the transitional information that the nurse and the attending physicians record on paper.

#### Step 2: Construct Hierarchical Task Analysis Process Maps

**Figure 1:**
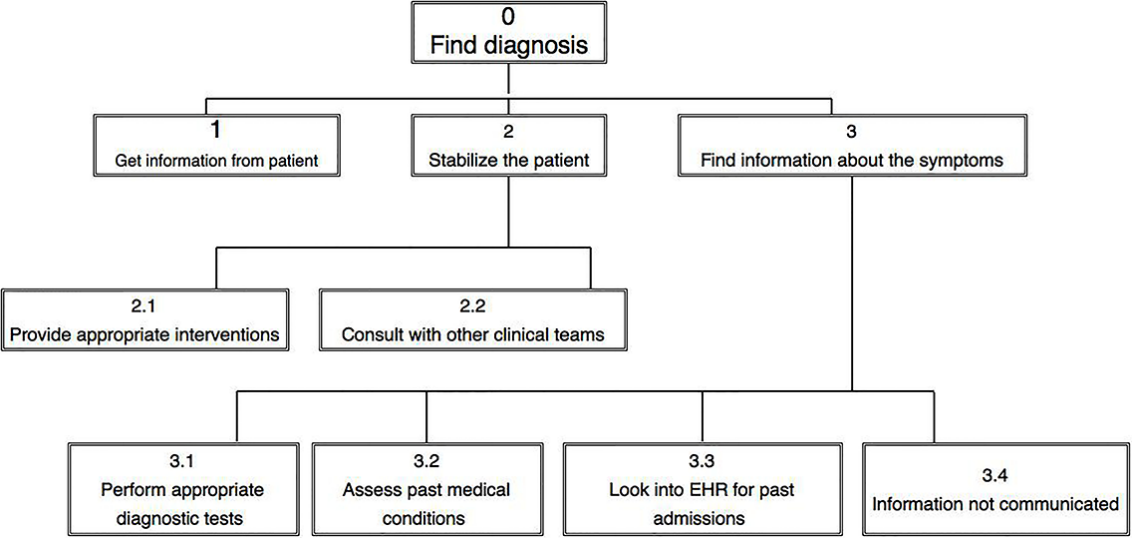
Hierarchical task analysis diagram: tasks and subtasks are designated by numbers. EHR: electronic health record.

The research team will analyze the observation transcript independently and construct HTA process maps for each case until there are no more tasks related to reach the diagnosis. We will merge the goals and tasks for the physician and the nurse to construct the process maps. For example, if the highest goal is *finding diagnosis*, we will merge nursing goal of *finding patients home medication history* as a subgoal under *finding diagnosis*. We will focus on the main goals associated with finding the correct diagnosis and represent the associated task steps to accomplish those goals in a hierarchical decision tree (Figure 1) [31,41,42]. After we have developed the HTA process maps for each of the 40 patient cases, we will validate the HTA process maps with 2 ED physicians [41,43]. Finally, we will map the failure-prone tasks’ steps from the decision tree, based on the list for detecting failures across the diagnostic process developed by the IOM committee, as described in Table 1 [44]. For example, if *consultation with other clinical team was not possible* (Figure 1, subtask 2.2), we will code that as information integration 4 (information from other team not available), or if *past medical conditions get missed* (Figure 1, subtask 3.2), then we will code that as information interpretation 1 (inaccurate interpretation of history). After mapping with failure-prone subtasks, we will start the HFMEA process.

**Table 1:** Nature of failures and description derived from the Institute of Medicine’s report.

Nature of failure	Failure description
Information gathering 1	Unable to elicit key information
Information gathering 2	Unable to get key history
Information gathering 3	Missed key physical findings
Information gathering 4	Failed to order or perform needed tests
Information gathering 5	Inappropriate review of test results
Information gathering 6	Wrong tests ordered
Information gathering 7	Tests ordered in wrong sequence
Information gathering 8	Technical errors in handling, labeling, and processing of tests
Information integration 1	Wrong hypothesis generation
Information integration 2	Inaccurate suboptimal weighing and prioritization
Information integration 3	Unable to recognize or weigh urgency
Information integration 4	Information from other teams not available
Information interpretation 1	Inaccurate interpretation of history
Information interpretation 2	Inaccurate interpretation of physical findings
Information interpretation 3	Inaccurate interpretation of test results
Establish explanation of diagnosis 1	Delay in considering diagnosis
Establish explanation of diagnosis 2	Patient develops infections or other complications
Establish explanation of diagnosis 3	Information missed to form hypothesis because of health information technology
Establish explanation of diagnosis 4	Signs and symptoms not recognized for specific disease
Establish explanation of diagnosis 5	Delay or missed follow-up

#### Step 3: Conduct Health Care Failure Mode and Effect Analysis

We will form a multidisciplinary ED team including 1 ED physician, 1 ED resident, and 1 ED nurse. We will then ask the team to conduct a brainstorming session with each HTA process map and discuss the vulnerable junctions (task steps) for patient safety, information loss, misinterpretation, group conflict, and factors associated with poor communication. The team will also discuss additional failure-prone task steps found in step 2 to find potential solutions. The team will rate the severity score (scale of 1 to 4) for each failure-prone task step as minor (score 1), moderate, major, and catastrophic (score 4). Then, the team will also rate the probability of the occurrence of such incidents on a scale of 1 to 4 as remote (score 1: happening rarely in 2 years), uncommon (once a year), occasional (every 3-6 months), or frequent (score 4: every month). We will combine the severity and probability scores to obtain a hazard score. We will focus only on subtasks with hazard scores of 5 or greater to identify potential solutions. Finally, the team will be asked to find potential solutions, including health IT interventions, that can improve the team communication and team diagnostic decision-making process. The final results will be shown as in Table 2 for each of the 40 patient cases.

Each brainstorming session will be limited to 50 min, will be audio recorded and transcribed, and will occur over multiple sessions. The principal investigator will conduct a final data analysis of the transcripts to identify the high failure-prone task steps and possible solutions.

**Table 2:** Factors contributing to failure in team-based diagnostic decision-making process.

Hazard score	Subtasks	Failure mode	Failure description	Causes	Effects	Remedial strategy
5	Subtask 2.2: consult with clinical teams	Information gathering 4	Information from other teams not available	Radiology is overwhelmed with tasks	Delay in patient diagnosis	Update radiology team to send urgent patient results first
7	Subtask 3.3: information overlooked in EHR^a^ for past admissions	Establish explanation of diagnosis 3	Information missed to form hypothesis because of health information technology	Information lost because of interruption	Wrong diagnosis	Actively engage different team members to focus on multiple data sources in EHR

**^a^**EHR: electronic health record.

#### Study Subjects and Recruitment Methods

We will recruit 4 ED physicians and 4 ED nurses for the observation study to increase provider diversity. For the HFMEA part of the study, we will recruit 2 ED physicians, 2 ED nurse, and 2 ED residents. A total of 14 providers will be recruited from 3 hospital sites by email and telephone, and a US $50 gift card will be provided for participation.

#### Sample Size Justification

On the basis of our pilot study sample size, we will observe 40 patient cases. We will include only adults (aged >18 years) for selecting cases. We will observe each scenario until the team reaches a consensus about the diagnosis. Previous studies have observed 32 to 50 cases for reaching data saturation [43,45-47].

#### Team Members Makeup

For this aim, we will assume the ED team includes the attending physician and the attending nurse. However, we will include senior and junior-level residents, radiology physicians, other nursing staff, pharmacists, and support staff based on the makeup of that current team on that particular shift.

#### Limitations

The HTA and HFMEA methods are time consuming, specifically observation, construction of the HTA, and data analysis. However, a 3-year timeline is reasonable. In addition, there may be concern that step 2 (HTA process maps) may not generate adequate failure-prone steps. However, step 3 (HFMEA) brainstorming session by the group will also identify failure-prone steps in addition to discussing failure-prone steps found in the HTA process maps and will complement each other.

### Aim 2: Understand the Barriers in Using Health Information Technology Tools for Team Collaboration

The research questions are as follows: (1) *What are the barriers to sharing information using current health IT tools?* and (2) *What are the leverage points* (ie, critical pieces of information that lead to a useful decision path [48]) *for the team during complex diagnostic decision-making tasks and negotiating conflict* [49]?

#### Aim 2 Methods Overview

We will conduct a Critical Incident Technique (CIT)–based team Cognitive Task Analysis (CTA) interview [50-53]. CTA is a process of understanding cognition while performing complex tasks. It provides a mechanism for eliciting and representing general and specific knowledge [54-56]. Team CTA is an extension of CTA that considers a team as a single cognitive entity (eg, more than a collection of individuals) [57,58]. CIT comprises a set of procedures for gathering facts on human behavior in a recent complex situation. In this study, team CTA will help us identify the barriers that ED team members face while gathering, integrating, interpreting information and forming hypotheses about the diagnosis using current health IT tools. Effective teamwork includes motivating and gathering information from each discipline, regardless of interdisciplinary conflicts [59].

#### Procedure

We will ask the team members to describe a recent complex case that was challenging to solve as a team for an admitted patient. Experiences related to critical incidents in interprofessional teamwork will be evoked by asking open-ended questions: “Are there any difficulties or challenges involved in working together using the current health IT tools?” followed by “Can you describe a situation that you remember in detail when you experienced such a difficulty?” Once the situation is established with time-specific detail, follow-up questions and probes will be asked to elicit the team’s dynamic decision-making strategies to negotiate conflicts, the specific actions by each team member, and the process by which the problem was solved. We will focus on how team members prioritize and rank patient information to negotiate conflict to reach consensus.

#### Study Subjects and Recruitment Methods

We will recruit 5 ED teams by email and telephone. Each team will consist of 4 clinicians, including 1 attending ED physician, 1 ED nurse, 1 ED resident, and 1 ED pharmacist. Inclusion criteria will be at least 1-year experience as a team member and a recent (within last 3 months) experience in working in the ED. Each clinician will receive a US $50 gift card for participation.

#### Data Collection

We will use the transcripts from the audio recordings of the interviews for data analysis. All patient identifiers will be removed.

#### Study Measures

The study measures are as follows: (1) cues and patterns of the team members’ preferences for using current health IT tools, (2) leverage points (cues related to shared and complementary cognition), (3) common sources of conflict and resolution strategies [60], and (4) complementary knowledge and skills to synthesize task elements.

#### Data Analysis

A total of 2 investigators will independently code the transcripts from the team CTA interviews and merge the individual codes into subthemes and later into broader themes through a process of negotiated consensus. We will code based on a qualitative content analysis process [61-63]. We will use ATLAS.ti software for data analysis.

#### Sample Size Justification

We will interview 20 providers for the team CTA interviews. Previous studies used a range of 6 to 30 providers for successfully conducting similar team CTA interviews [64-67].

#### Limitations

CTA studies are based on memories. It can be difficult to explore past information, as key pieces of information may not be stored properly in the memory [68]. Therefore, we conducted a pilot study to prepare the questions that can evoke the response needed for data analysis [55].

### Aim 3: Design and Evaluate a Collaborative Decision-Making Prototype

#### Aim 3 Methods Overview

We will develop complex case vignettes, design the prototype, and conduct the usability study.

#### Complex Team-Based Diagnostic Case Vignette Design

We will design 8 complex clinical vignettes based on team-based diagnostic problems from our findings from aims 1 and 2 [69]. We will validate the complexity of cases with 3 ED physicians. These cases will be presented to participants in a mock electronic EHR.

#### Prototype Design: Preliminary Design Concept

**Figure 2:**
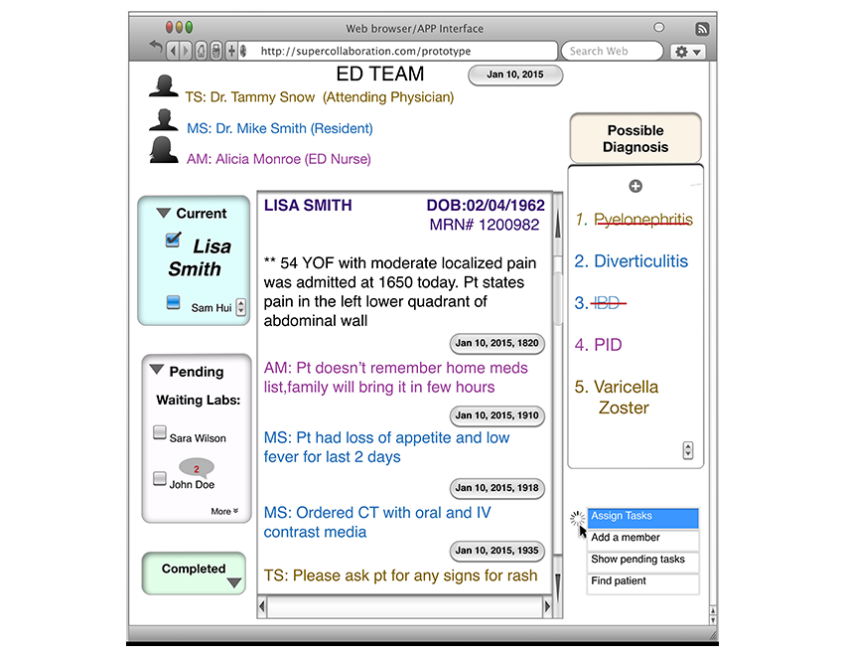
Screenshot of the mock-up user interface for the collaborative decision-making prototype.

The purpose of this prototype is to gather, integrate, and collect vital patient information from different team members to rank and filter information for making an informed diagnostic decision collaboratively. The results from aim 1 will inform design by allocating failure-prone task steps as the main focus in the interface (ie, if *unable to get key history* becomes a major failure-prone task, then a separate tab should be created in the interface as *pending information for patients*). The results from aim 2 will provide specific design allocation for features such as knowledge characteristics (ie, team should be able to see updates of all patients in 1 screen) or expertise process requirements (ie, comments from each team member based on medical expertise should be grouped to improve trust in the information) and so on. For example, in this shared platform (Figure 2), all team members can enter relevant information regarding the patient (color coded as mocha for ED physicians, blue for residents, and magenta for nurses). Everyone can also add possible hypotheses about the diagnosis in the *possible diagnosis* tab. Only the ED physician will be able to delete a diagnosis (shown as red strike-through in the diagnosis tab). Physicians and other team members can also assign tasks and group patients by *waiting labs* or *completed* (left side of the interface). This is an initial version only. The design will be refined based on aim 1, aim 2, and iterative design in aim 3 to ensure patient safety.

#### Iterative Design

To facilitate rapid development, initial low-fidelity mock-ups and storyboarding will be iteratively created to illustrate the design and functionality of the tool and load it in a laptop. We will use the usability inquiry approach for the iterative design to understand user’s likes, dislikes, and needs [70]. The interprofessional research team (including 9 clinicians with diverse clinical background and 6 researchers) will then iteratively review and revise the mock-up based on the written and verbal feedback related to usability (think-aloud methods), efficiency, and ease of use for 3 months or until no further revisions are identified. Think-aloud methods will provide rich verbal data about specific changes and functionalities of the initial mock-up [71-73]. We will audio record and screen record (using Camtasia Studio) the sessions to analyze verbal feedback and measure the mouse movements. We will analyze the data using think-aloud methods and screen recordings to identify design issues and iterate interface functionalities accordingly.

#### Usability Testing of the Prototype

We will conduct the study in the Emanate Health System. We will provide initial training to each provider about the scope of the research, the prototype tool, and the 3 steps of usability testing that will reveal the prototype’s ease of use, familiarity, effectiveness, and user satisfaction. Each session will last less than 60 min. We will conduct the usability testing of the prototype in the following 3 steps:

- Step 1: evaluate ease of use and familiarity
- Step 2: test prototype effectiveness
- Step 3: conduct prototype evaluation

### Step 1: Evaluate Ease of Use and Familiarity (10-12 Min)

We will use the *cognitive walkthrough* evaluation method to understand the user’s background and the level of mental effort [74-76]. First, we will ask each provider about his or her initial perception and what action each of the interface components (eg, buttons and checkboxes) is expected to perform when interacted with. Then we will ask each provider to complete a sequence of tasks and subtasks while using the prototype and will provide assistance when asked. An example of a potential task is as follows: “Please use the interface to add a potential diagnosis” or “Please assign a task to your colleague.” Providers will then be given 5 min to use the tool on their own to gain familiarity, and any questions asked will be answered. Finally, we will ask the providers to conduct similar tasks without assistance to understand familiarity and ease of use. The number of times assistance is needed will be audio recorded and will serve as a descriptive measure of ease of use for data analysis. The ability of providers to accomplish tasks without assistance will serve as a marker of high ease of use. The principal investigator will conduct the final data analysis from the audio transcripts to find the number of times assistance was required before and after demonstration.

### Step 2: Test Prototype Effectiveness (36 Min)

To measure the effectiveness of decision making using the prototype*,* we will use a 2 randomized between (presence/absence of the prototype) × 2 between (expertise) × 2 within (time pressure) factorial design. Each team will receive 8 vignettes presented in random order. The main effect of the presence or absence of the prototype tests the experimental question. With this design, we are also able to test for the interaction between the impact of the interface and the domain of expertise under time pressure. For example, the interface could change the interaction between time pressure and domain expertise (a 3-way interaction), eliminate the influence of time pressure overall for both ED expert and non-ED expert teams (2-way interaction), and have a main effect on quality for everyone in all conditions.

### Step 3: Conduct Prototype Evaluation (10-12 Min)

We will conduct a team satisfaction survey to understand team members’ *satisfaction level* and System Usability Scale survey to understand the *ease of use* with the prototype. First, we will ask each provider to complete a Web-based user satisfaction survey to measure individual team members’ satisfaction for using the prototype [77]. This teamwork process–specific survey focuses on organizational context, team task design, information sharing, and team processes [78]. Research has shown that it is difficult to capture team-specific activities through commonly used surveys such as National Aeronautics and Space Administration Task Load Index [29]. This survey (6-point Likert scale) has been used for understanding team dynamics in *other successful fields* when evaluating group decision support tools [79-85]. Data analysis will include factor analysis, scale reliability analysis, aggregation analysis, and path analysis [77]. Finally, the ease of use of the prototype will be evaluated using the System Usability Scale, a rapidly administered, 10-question, 100-point scale designed to evaluate a user’s subjective assessment of usability [86]. Data analysis will include total score calculations based on the participants’ answers.

#### Dependent Variables

We will have 2 dependent variables, diagnostic accuracy and overall team diagnostic decision quality. For *diagnostic accuracy*, the presence of the correct diagnosis in the top 3 items of the diagnostic differential will be computed as a dichotomous (yes or no) variable. For example, if the clinical team correctly diagnoses the top 2 of the 3 diagnoses in the vignettes, it will be counted as *yes*. The *overall team diagnostic decision quality* will be an aggregate score created from the combination of the (1) correct diagnosis, (2) rating of the confidence of the final diagnosis (on a scale of 0-3, with 0 being the lowest confidence rating), and (3) correctly ordered diagnostic tests. The overall score will range from 0 to 10, with correct diagnoses receiving 4 points and confidence ratings and correctly ordered tests receiving 3 points each.

#### Independent Variables

Time pressure is an independent variable because we will be assigning *high time pressure* as less than 3 min and *low time pressure* as less than 6 min.

#### Procedures

We will explain the procedure and ask participants to finish 4 cases under high time pressure (<3 min) and 4 cases under low time pressure (<6 min). Initially, all team members, the nurse, the resident, and the physician, will be distant and reviewing the case independently. They will use the decision-making prototype (loaded in laptops) to communicate among themselves for sharing information to establish an explanation for the diagnosis. They will have the final 1 min to discuss, as a group, the high time pressure cases and the final 2 min for low time pressure cases to reach consensus about the correct diagnosis. We will ask each team to rate their confidence in the diagnosis. We will also note the responsible team members who voice their concerns regarding each of the complex patient cases.

#### Data Analysis

We will use Chi-square test to evaluate association between the independent variables with the decision quality. We will use analysis of variance (ANOVA) to calculate the mean difference within and between ED expert teams’ and non-ED expert teams’ decision quality. The within- and between-group design will provide us with a sample size adequate for an ANOVA test. The proportion of decisions made with the correct diagnosis and overall decision quality will be shown as a percentage value using ANOVA. If the distribution is not normal, we will use the General Linear Model for the data analysis [87].

#### Overall Study Measures

The overall study measures are as follows: (1) providers’ comments about the initial design, (2) number of times assistance was required before and after demonstration, (3) scores for team decision quality, and (4) survey responses.

#### Study Subjects and Recruitment Methods

We will recruit 12 teams with each team (6 ED experts and 6 non-ED experts) comprising a physician, a resident, and a nurse (36 providers: US $50 gift card will be provided) by emails and phone calls. The inclusion criterion for the ED team is that members should have at least 6 months’ experience working in the ED, and non-ED teams should include providers with expertise in other clinical domains.

#### Sample Size Justifications and Power Calculation

Previous studies successfully enrolled 7 to 36 providers for similar usability studies [74,88-93]. The 12 teams and 8 case vignettes in this within- and between-group design with a 2-tailed alpha of .05 and a moderate effect size give a power of 0.83.

#### Limitations

Reasonable efforts will be made to ensure the prototype realistically simulates a shared workspace for team collaboration. However, the assessment provides initial steps in understanding team diagnostic decision quality, serving as a foundation for future study in real-world situations.

## Results

We are collecting preliminary data for this study between the period of 2019 and 2020. The results are expected to be published between 2020 and 2021.

## Discussion

### Collaborative Decision Support Design

Studies have shown that uneven information can result from the exclusion of team members from messages or the failure of team members to share uniquely held information [94-96]. Studies also show that task conflict can arise when some team members operate with incomplete information, suggesting that when information is provided, agreement can quickly be reached [97-99]. Collaborative decision support tools have proven to be effective in *other successful fields* in resolving conflict by providing a platform to coordinate team tasks.

This protocol addresses the problem of diagnostic error through innovative approaches for reducing the loss of vital patient information and effectively sharing key information to form correct diagnosis as a team. The robustness of the methodology used in this protocol has been applied to other successful fields. Observation, HTA (aim 1), and team CTA (aim 2) methods have been applied in military, naval warfare, aviation, air traffic control, emergency services, and railway maintenance [100-105]. This Web-based prototype, in the long term, can be integrated with EHR as well as installed in mobile (app-based) devices for providers to capture the transitional information and share this information with team members to reach the correct diagnosis. For this protocol, we are exploring the prototype only as front end; it will not be integrated or installed into any systems or any EHR. The protocol is planned over a period of 5 years. The research team is experienced and plans to execute the project before the timeline.

### Conclusions

The results from this study can shed light on improving diagnostic decision making by incorporating diagnostics rationale from team members. We believe a positive direction to move forward in solving diagnostic errors is by incorporating all team members, and using informatics.

## Abbreviations

ANOVA: analysis of variance
CIT: Critical Incident Technique
CTA: cognitive task analysis
ED: emergency department
EHR: electronic health record
HFMEA: health care failure mode and effect analysis
HTA: hierarchical task analysis
IOM: Institute of Medicine
IT: information technology
